# Balanced: a randomised trial examining the efficacy of two self-monitoring methods for an app-based multi-behaviour intervention to improve physical activity, sitting and sleep in adults

**DOI:** 10.1186/s12889-016-3256-x

**Published:** 2016-07-30

**Authors:** Mitch J. Duncan, Corneel Vandelanotte, Stewart G. Trost, Amanda L. Rebar, Naomi Rogers, Nicola W. Burton, Beatrice Murawski, Anna Rayward, Sasha Fenton, Wendy J. Brown

**Affiliations:** 1 School of Medicine & Public Health; Priority Research Centre for Physical Activity and Nutrition, Faculty of Health and Medicine, The University of Newcastle, University Drive, Callaghan, NSW 2308 Australia; 2 School of Human Health and Social Science; Physical Activity Research Group, Central Queensland University, Building 18, Bruce Highway, Rockhampton, QLD 4702 Australia; 3 Institute of Health and Biomedical Innovation, School of Exercise and Nutrition Sciences, Queensland University of Technology, Brisbane, QLD 4059 Australia; 4 Sydney Medical School, The University of Sydney, Sydney, NSW 2050 Australia; 5 School of Human Movement and Nutrition Sciences, The University of Queensland, St Lucia, QLD 4072 Australia

**Keywords:** Physical activity, Sedentary, Sleep, App-based intervention, Adults

## Abstract

**Background:**

Many adults are insufficiently physically active, have prolonged sedentary behaviour and report poor sleep. These behaviours can be improved by interventions that include education, goal setting, self-monitoring, and feedback strategies. Few interventions have explicitly targeted these behaviours simultaneously or examined the relative efficacy of different self-monitoring methods.

**Methods/Design:**

This study aims to compare the efficacy of two self-monitoring methods in an app-based multi-behaviour intervention to improve objectively measured physical activity, sedentary, and sleep behaviours, in a 9 week 2–arm randomised trial. Participants will be adults (*n* = 64) who report being physically inactive, sitting >8 h/day and frequent insufficient sleep (≥14 days out of last 30). The “*Balanced*” intervention is delivered via a smartphone ‘app’, and includes education materials (guidelines, strategies to promote change in behaviour), goal setting, self-monitoring and feedback support. Participants will be randomly allocated to either a device-entered or user-entered self-monitoring method. The device-entered group will be provided with a activity tracker to self-monitor behaviours. The user-entered group will recall and manually record behaviours. Assessments will be conducted at 0, 3, 6, and 9 weeks. Physical activity, sedentary behaviour and sleep-wake behaviours will be measured using the wrist worn Geneactiv accelerometer. Linear mixed models will be used to examine differences between groups and over time using an alpha of 0.01.

**Discussion:**

This study will evaluate an app-based multi-behavioural intervention to improve physical activity, sedentary behaviour and sleep; and the relative efficacy of two different approaches to self-monitoring these behaviours. Outcomes will provide information to inform future interventions and self-monitoring targeting these behaviours.

**Trial registration:**

ACTRN12615000182594 (Australian New Zealand Clinical Trials Registry. Registry URL: www.anzctr.org.au; registered prospectively on 25 February 2015).

## Background

Physical activity and sleep are important for the promotion of health and well-being [1–3]. Insufficient moderate-to-vigorous intensity physical activity, prolonged sedentary behaviour, and poor sleep behaviours - sleeping either too few/many hours or having poor quality sleep – increase the risk of cardiovascular disease (CVD), type 2 diabetes, poor quality of life, anxiety, depressive symptoms, and all-cause mortality [2–7]. Internationally, considerable proportions of the adult population report engaging in one or more of these health compromising behaviours [8–13].

Although multi-behaviour change interventions are frequently conducted, few explicitly target improvements in physical activity, sedentary and sleep behaviours [14–16]. There is good rationale to target these behaviours simultaneously, as they are thought to share a reciprocal relationship. Greater activity levels are thought to improve sleep quality, and better sleep quality can contribute to higher levels of moderate-to-vigorous intensity physical activity and total daily activity [17–20]. In addition, these reciprocal relationships may be enhanced through co-action - a mechanism in multi-behaviour change interventions where change in one behaviour can lead to change, either intentionally or unintentionally, in subsequent behaviours [21]. To maximise change in multi-behaviour interventions it is necessary to provide participants with dedicated behaviour change techniques specific to the targeted behaviours such as goal setting, self-monitoring, and feedback on performance [22–24]. There is substantial evidence that self-monitoring improves a number of health behaviours [25, 26]. Self-monitoring involves individuals tracking behaviour, evaluating progress towards a pre-determined standard, and being aware of the factors that inhibit or facilitate progress [23, 24, 27]. Physical activity, sedentary and sleep behaviours can be manually self-monitored by individuals recalling key aspects of the behaviour (e.g., duration of activity or sleep, time to sleep, time to wake) and recording this information into a paper or electronic diary [28–32]. However, this is vulnerable to social desirability and recall bias, and can have high participant burden [33]. Previous interventions have included devices such as pedometers to partially overcome these limitations by asking participants to enter pedometer steps into the intervention platform [33]. Self-monitoring can also now be automated. This can be achieved by using newly available activity trackers (eg. Fitbit) that also allow physical activity, sedentary and sleep behaviours to be self-monitored and automatically synchronised to mobile devices, which can display feedback on behaviour [25, 34]. However, these devices may increase costs of intervention, present additional technical barriers (e.g. synchronising data across multiple platforms) and the also lower some of the cognitive processing associated self-monitoring which is a key part of self-monitoring [27]. The impact of this on the efficacy of self-monitoring interventions is unknown.

Technology-based interventions such as those delivered by websites and smartphone applications ‘apps’ are increasingly used due to the large potential reach they offer, increased access, including the ability to overcome the need to attend face to face sessions to receive the intervention [35–37]. Technology-based interventions can integrate key behaviour strategies, including self-monitoring and feedback, and to date have been successful in improving a variety of health behaviours over shorter time periods (<12 weeks) [26, 36, 37]. Participant usage of and engagement with the intervention platform is frequently examined and appears to be related to intervention efficacy [26, 36, 38]. Though many technology-based interventions observe large declines in usage and engagement during the first four weeks of the intervention period and do not assess behaviour until much later (e.g., week 12) [28, 38, 39]. Examining behaviour change during time periods when usage and engagement frequently declines may provide greater insight on this relationship and improve subsequent interventions.

The primary aim of this study is to compare the efficacy of device-entered and user-entered self-monitoring methods in a app-based multi-behaviour intervention to improve objectively measured physical activity, sedentary, and sleep behaviours, in a 9 week 2-arm randomised trial. Secondary outcomes will include self-reported physical activity, sitting time, sleep quality, sleep hygiene, anxiety, depression, stress, quality of life and cardiometabolic risk. Potential mediators include psychosocial correlates of behaviours and intervention usage and engagement. Process measures will be collected at the conclusion of the 9 week intervention period and will include reasons for participating in the study, expectations of the study, perceived aesthetics, functionality and appropriateness of the intervention platform, and suggested modifications to the intervention.

## Methods

### Trial design

The Balanced study is a two-arm randomised trial over 9 weeks. The intervention groups are:

1. *Balanced* intervention with device-entered self-monitoring method,
2. *Balanced* intervention with user-entered self-monitoring method.

**Fig. 1:**
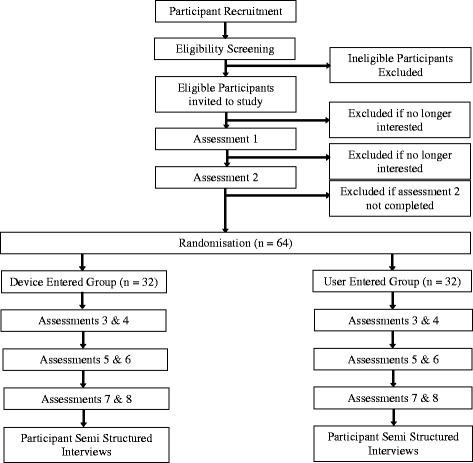
Study Design

All participants will have access to the same smartphone-based *Balanced* intervention only the method of self-monitoring is different. Participants in the device-entered self-monitoring group will be provided with a wrist-worn activity tracker to automatically measure and upload physical activity, sedentary and sleep behaviour data. Participants in the user-entered self-monitoring group will recall and manually enter information on physical activity, sedentary and sleep behaviours. Participants will complete assessments at 0, 3, 6, and 9 weeks (Fig. 1). The study received approval from the Human Research Ethics Committee of The University of Newcastle, Australia (Reference Number H-2014-0336) and is registered with the Australian and New Zealand Clinical Trials Registry (ACTRN12615000182594). All participants provided informed consent to participate and could withdraw at any time for any reason. The funding body had no role in the design, conduct or reporting of the trial.

### Participant recruitment

Sixty-four participants will be recruited from the Newcastle area, New South Wales, Australia using posters distributed at The University of Newcastle Callaghan campus, local businesses, community notice boards (both physical and electronically), community events; and study advertisements via radio, electronic communication (email lists, social media), and participant registries. Recruitment commenced in July 2015. Individuals interested in participating in the trial will be directed to an online screening survey to assess eligibility. Those who meet the inclusion criteria will be contacted by project staff via phone or email to arrange an appointment time. Ineligible participants will be contacted via phone or email to advise them they are ineligible and offered access to the Balanced app.

### Eligibility

To be eligible for inclusion into the study, individuals must report:

1. Being aged between 18 and 55 years,
2. Weight and height consistent with a Body Mass Index (BMI) between 18.5 and 35.0,
3. Engaging in less than 30 min of moderate to vigorous intensity physical activity on 5 days per week which is comparable with recommendations [40],
4. Spending at least 8 h per day sitting on 5 or more days per week, which is comparable with research identifying risk levels of mortality and chronic disease above this level [41, 42],
5. Waking up feeling like they needed more rest/sleep on 14 or more days per month, which is comparable with research identifying higher risk of chronic disease above this level [4],
6. Absence of any condition that makes it unsafe for them to change their activity, sitting and sleep behaviours,
7. Absence of an existing sleep disorder such as insomnia, sleep apnoea, restless legs syndrome,
8. Not being employed in shift work,
9. Not taking any medications to induce sleep,
10.  Not travelling to any destination requiring a change in time zone of more than 3 h during the intervention period.
11.  Not currently using an app or activity tracker to track physical activity, sedentary or sleep behaviours.
12.  Having access to either an Android or iOS smartphone or tablet with access to the internet.

### Study procedure

Eligible participants will be asked to attend the University of Newcastle on eight occasions to complete four assessments (2 visits required per assessment). Table 1 provides an overview of the assessments conducted at each visit. Visits 3, 5 and 7 occur 3, 6 and 9 weeks after visit 2. At visits 1, 3, 5, and 7 participants will be provided with an accelerometer to wear continuously for 7 days and return the accelerometer 8 days later visits 2, 4, 6 and 8 respectively, no other assessments occur at visits 1, 3, 5 and 7.

**Table 1:** Overview of measurement tools, and timing of measurements

Outcome	Measure	Assessment point (Visit)
Primary Outcomes		
Objectively Measured Physical Activity, Sedentary Behaviour, Sleep^a^	Geneactiv Accelerometer	2, 4, 6, 8
Secondary Outcomes		
Self-Reported Physical Activity, Sitting Time, Sleep Quality, Sleep Timing	Active Australia Survey [66–68]	2, 4, 6, 8
	Workforce Sitting Questionnaire [69]	2, 4, 6, 8
	Pittsburgh Sleep Quality Index [70]	2, 4, 6, 8
	Sleep Timing Questionnaire [72]	2, 4, 6, 8
	Behavioral Risk Factor Surveillance Sleep Module [71]	2, 4, 6, 8
*Depression, Anxiety and Stress*	Depression, Anxiety and Stress Scale [73, 74]	2, 4, 6, 8
*Health related quality of life*	Centres for Disease Control Healthy Days Instrument [78–80].	2, 8
Mediators		
*Lifestyle Behaviour Habit*	Automaticity subscale of self-report behavioural automaticity index (Physical Activity) [81]	2, 4, 6, 8
	Automaticity subscale of self-report behavioural automaticity index (Sitting) [81]	2, 4, 6, 8
	Automaticity subscale of self-report behavioural automaticity index (Sleep) [81]	2, 4, 6, 8
Sleep Hygiene	Sleep Hygiene Index [82]	2, 4, 6, 8
*Social Cognitive Factors*	Social Cognitive Factors (Physical Activity)	2, 4, 6, 8
	Social Cognitive Factors (Sitting)	2, 4, 6, 8
	Social Cognitive Factors (Sleep)	2, 4, 6, 8
*Usability, Satisfaction*	System Usability Scale [84]	8
	Satisfaction. Newly developed items	8
Process Evaluation	Semi-Structured Interview	8
Engagement and Usage	App database	Continuous
Demographics	Commonly used items	2
Presenteeism	World Health Organization Health and Work Performance Questionnaire [85, 86]	2, 8
Anthropometrics and Blood Pressure	Standard Protocols for Height, Weight, Waist Circumference, Blood Pressure [87]	2, 4, 6, 8
Cardiometabolic Risk	Cardiochek PA measures of total cholesterol, HDL cholesterol, LDL cholesterol, triglycerides and glucose	2, 8

^a^The Geneactiv is provided to participants to begin wearing at visit 1, 3, 5, and 7 respectively

The research assistant performing height, weight, waist circumference and blood pressure measures received training to perform these measures and will be blinded to participant group allocation. Participants will also be provided with a gift voucher to the value of $10 at the completion of each of assessments in visits 2, 4, 6, and 8. Following completion of measurements during visit 2 participants will be randomised to one of the intervention groups and provided with access to the *“Balanced”* app; participants allocated to the Device-entered self-monitoring group will be provided with a Fitbit (Charge HR) activity tracker.

### Intervention

“*Balanced*” is multi-behavioural intervention to increase time spent being physically active, decrease sedentary time and improve sleep quality. Intervention strategies include the provision of educational materials (e.g. health benefits of target behaviours, National guidelines on each behaviour, behaviour change strategies, creating action plans, sleep hygiene), goal setting and daily self-monitoring. Participants were not required to use the app for any specified time period however were instructed that it was designed for daily monitoring of behaviour and that they could use it as little or as much as they preferred. No prompts were provided to use or engage with the intervention. The intervention is able to be continuously accessed throughout the intervention and assessment periods and is accessible through a specifically designed mobile device app, available on both Android and iOS operating systems. The usability of the platform has been previously evaluated using a think aloud methodology similar to other research [43].

The development of the intervention platform was guided by operationalising constructs from social cognitive and self-regulatory theories (e.g., *education, goal setting, self-monitoring, feedback on behaviour)* which are consistently identified as important drivers of behaviour change [44–47]. The intervention platform has five sections as detailed below consistent to both intervention groups: 1) *Dashboard*, 2) *Your Stats* 3) *Progress*, 4) *Resources* and 5) *My Profile*.

### Dashboard

**Fig. 2:**
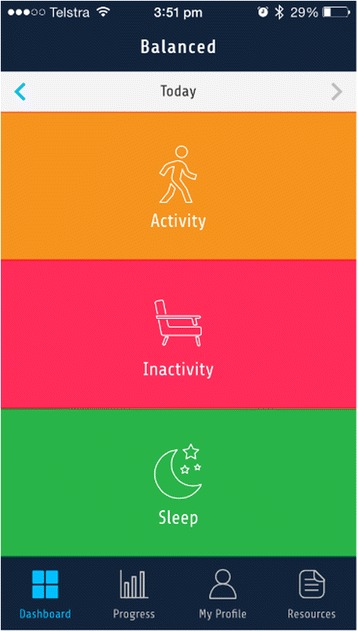
Screenshot of the Balanced Dashboard displaying Orange, Red and Green feedback to the user on Activity, Sedentary and Sleep behaviours respectively

The *Dashboard* section (Fig. 2) uses a traffic light system to provide participants with a visual representation of their progress toward achieving a predetermined standard level of each behaviour. These levels were selected based on guidelines or available evidence on the lowest risk of overall mortality or cardiovascular disease [1, 2, 48]. The traffic light colours change when the information entered in the *Your Stats* section is updated (user-entered group) or when the Fitbit is synchronised (device-entered group). Green reflects that a user’s behaviour meets or exceeds the predetermined standard. Orange reflects that a user’s behaviour somewhat lowers their risk, however more positive changes are needed. Red reflects that a participant’s behaviour is markedly below the predetermined standard and associated with significant health risk. The dashboard is intended to operationalise the construct of feedback on behaviour.

### Your stats

**Fig. 3:**
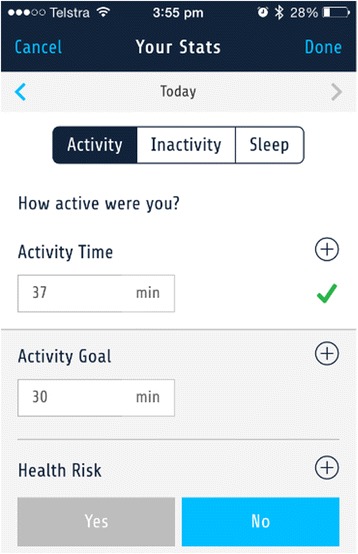
Screenshot of Your Stats section for Activity

The *Your Stats* section (Fig. 3) allows participants to see time spent in moderate-to-vigorous intensity physical activity, time spent sedentary, time to sleep and time to wake, and sleep quality. The user-entered group used this section to enter data on each of these behaviours. In this section, participants can enter goals for all behaviours except sleep quality because individuals can engage in activities that can promote improved sleep quality (eg. sleep hygiene behaviours), but sleep quality is not directly under their control. Participants may also enter a subjective rating to indicate whether they believe their current behaviour is a risk to their health, using a dichotomous yes or no response format, in this section. This seeks to engage participants in evaluation of their behaviour, in relation to the information provided in the resources section and feedback provided by the traffic light system. It is intended to align their perceptions of their behaviour with their actual level of behaviour, as many people are likely to have misaligned perceptions of their lifestyle behaviours [49, 50]. The information entered in this section is intended to operationalise the constructs of goals setting and self-monitoring.

### Progress

**Fig. 4:**
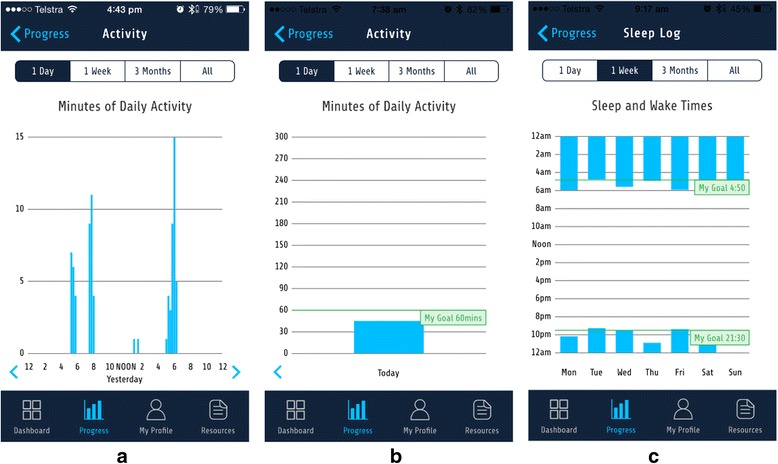
**a** Daily summary graph of activity for the device-entered group. **b** Daily summary graph of activity for user-entered group. **c** Graph of time to sleep and time to wake over a 1 week period

The *Progress* section provides graphical feedback of behaviours in comparison to goals on duration of physical activity, sedentary behaviour and sleep, sleep quality, and the pattern of sleep (time to sleep and time to wake), using four time periods: daily, weekly, 3 month, and total usage period. The device-entered group are provided with graphs displaying summaries of the number of minutes spent in moderate-to-vigorous intensity physical activity, and in sedentary behaviour, in 15 min periods (See Fig. 4a). The manual-entered group receives daily summaries for these behaviours in the form of a single bar graph (See Fig. 4b). The sleep pattern graph shows the time to bed and time to wake, as consistency in these times is a key behavioural target for improving sleep quality (Fig. 4c) [12, 51, 52].

### Resources

The *Resources* section provides information on the benefits of, and barriers to improving targeted health behaviours, and details of the criteria used to determine colours in the Traffic Light system on the Dashboard. Strategies to improve behaviours were adapted from our previous interventions and existing resources (i.e. National Guidelines, National Heart Foundation, Sleep Health Foundation), including goal setting strategies [28, 40, 51, 53, 54]. Sleep education materials will include sleep hygiene education information based on existing information [51, 53]. Education materials will promote the *formation of habits* around target behaviours (i.e. scheduling activities, creating routines around behaviours) to engrain behaviours in daily life and promote longer term changes [55]. These materials use an approach similar to that used in previous interventions, by providing concise information on each behaviour in a “Why”, “How”, and “How much” format [28]. The resources section is intended to operationalise the behaviour change techniques of education and action planning.

### My Profile

The *My Profile* section allowed participants to alter their email address, change their password and provided a description of the research team.

### Device-entered self-monitoring

**Fig. 5:**
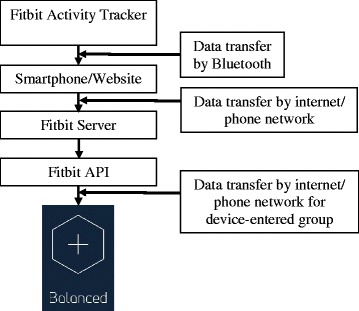
Data transfer and synchronisation between Fitbit Activity Tracker, Smartphone/Website, Server and the Balanced app

Participants in the device-entered group will self-monitor physical activity, sedentary and sleep behaviour using data from the Fitbit. The Fitbit measures these behaviours and automatically synchronises with the Balanced platform using the Fitbit Application Programming Interface (API) (See Fig. 5) to display information on these behaviours in the Balanced platform. Participants will not be required to interact with the Fitbit website or app in anyway, furthermore they will be asked not to use these throughout the intervention. In the device-entered group, activity and sedentary behaviour are measured using steps per minute criteria of >100 steps and zero steps per minute respectively [56–58]. Time to sleep, time to wake and sleep quality (derived from the ratio of sleep duration/time between time to sleep and time to wake) will be derived from the Fitbit data. Sleep quality is subsequently classified into a 5 point scale where 1 is lower sleep quality and 5 is higher sleep quality [59].

### User-entered self-monitoring

Participants in the user-entered group will self-monitor physical activity, sedentary, and sleep behaviour by manually entering this into the intervention platform using the My Stats section (Fig. 3). The user-entered group will be asked to self-monitor time spent in moderate-to-vigorous intensity physical activity, which is defined based on increases in breathing and heart rates and increased difficulty to carry a conversation and talk. The user-entered group will be asked to self-monitor their sedentary behaviour based on time spent sitting. The user-entered group will be asked to enter the time they went to sleep and woke up, and to self-monitor sleep quality using a 5 point scale from 1 (lowest quality) to 5 (highest quality) based on their perceived quality of their sleep.

### Randomisation

Participants will be randomised to one of the two intervention groups by a researcher not involved in participant assessments, after completion of their baseline assessment (Visit 2). The randomisation sequence will be generated using a computer-based random number generator using blocks of 4 and 6 [60]. The sequence will be generated by a researcher not involved in participant assessments and will be stored on a central database not accessible to those completing the assessments. Group allocations will be concealed in sequentially numbered opaque envelopes.

### Outcome measures

#### Primary outcome

##### Activity, sedentary and sleep behaviour

The Geneactiv is a small (36 × 30 × 12 mm, 16 g) waterproof accelerometer which has been shown to provide valid estimates of physical activity, sedentary and sleep behaviours [61, 62]. Participants will be asked to wear the Geneactiv activity monitor 24 h per day for 7 days on their non-dominant wrist and complete a written monitoring log to record the time of day that they go to bed, wake up, arrive at and leave work (if employed) and if the monitor was removed for any reason. Throughout the assessment period participants will receive text message reminders to wear the activity monitor approximately every 3 days.

The monitors will be set to collect data at 40-Hz. For data to be included in the analyses, a minimum of 5 days wear time, with at least 16 h wear per day will be required [63]. Daily time spent in sedentary, light and moderate-to-vigorous intensity physical activity (MVPA) will be determined using the Geneactiv data and the acceleration intensity thresholds developed by Hildebrand et al. [62]. In addition, the duration of specific activity types representing sedentary behaviour (i.e. sitting, standing stationary) and moderate-to-vigorous intensity physical activity (i.e. brisk walking, running) during waking hours will be quantified using the Random Forest activity classifier developed by Pavey and colleagues [64]. Geneactiv data will be used in combination with log data on time to bed and time to wake, to provide Geneactiv derived estimates of sleep onset and offset each day, sleep duration, and nightly awakenings using the R-package “GGIR” [61]. The ratio of sleep duration and time between sleep onset and offset will be used as an indicator of sleep efficiency. The within-participant standard deviation in sleep onset and offset will be calculated to provide a measure of variation in sleep-wake behaviour [65]. The primary outcomes of daily minutes of moderate and vigorous intensity physical activity, sedentary behaviour, sleep efficiency and sleep-wake variability will be averaged across days that satisfy minimum wear time criteria.

### Secondary outcomes

#### Self-reported physical activity, sitting, sleep

Self-report measures of physical activity, sitting and sleep are collected for several reasons including in the event that compliance with the accelerometer protocol is low or device malfunction. Self-reported sitting time can provide insight the domain of sitting that changes occur in and many sleep interventions report changes in self-reported sleep quality allowing comparison to these studies. The Active Australia survey will be used to assess the frequency and duration of self-reported walking for recreation and transport (combined), moderate and vigorous intensity physical activity over the last week. It has demonstrated acceptable levels of test-retest reliability (k = 0.50) and validity (k = 0.26–0.46) in population based surveys and is sensitive to detecting changes in physical activity in interventions [66–68].

The Workforce Sitting Questionnaire will be used to assess self-reported sitting time [69]. This instrument assesses the time spent sitting at work, watching TV, using a computer at home, transport and during other leisure activities on work and non-work days. It has acceptable levels of test-retest reliability (ICC = 0.46 – 0.90) and criterion validity compared to waist worn accelerometry (*r* = 0.18 – 0.46) [69].

Self-reported sleep quality will be assessed using the Pittsburgh Sleep Quality Index (PSQI) and the Behavioral Risk Factor Surveillance Sleep Module [70, 71]. The PSQI assesses the duration and quality of sleep over the previous month using 19 items that assess seven separate components of sleep, including duration, sleep latency and sleep problems. Each of the seven components are scored from zero to three and are summed to provide an overall score of sleep quality ranging from 0 to 21 where higher scores indicate poorer sleep quality. The Behavioral Risk Factor Surveillance Sleep Module contains 5 items assessing the average duration of sleep in a 24-h period, number of days in the previous month that an individual reports feeling they did not get enough rest or sleep, and unintentionally falling asleep during the day. The instrument also assesses if a person nodded off or fell asleep, even just for a brief moment, while driving in the last 30 days (yes, no, not applicable) and if they have ever been told they snore (yes, no, don’t know, unsure) [71]. Self-reported sleep timing and variation in sleep timing will be assessed using the 18 item Sleep Timing Questionnaire [72]. This instrument assesses the earliest and latest time of day a person usually goes to bed and wakes up, the usual time of day a person goes to bed and wakes up and the variation in these times on both weekends and week days. The Sleep Timing Questionnaire has demonstrated acceptable levels of test-retest reliability (*r* = 0.70), validity with accelerometery (*r* = 0.59) and sleep diary (*r* = 0.83–0.86) measures of sleep timing.

#### Depression, anxiety and stress

Physical activity, sedentary and sleep behaviours are associated with symptoms of depression, anxiety and stress and participants will complete the Depression, Anxiety and Stress Scale (DASS-21) to assess these [73–77]. Each of the 21 items in this scale asks participants to report how much each emotional experience (e.g., over reacting, feeling sad/depressed, feeling scared for no reason) statement applied to them over the previous week, using three response options from “did not apply to me at all” to “applied to me very much, or most of the time” [73, 74].

#### Health related quality of life

Health related quality of life will be assessed using the valid and reliable Centres for Disease Control Healthy Days Instrument [78–80]. This instrument assesses the self-rated health, frequency of physical and mental unhealthy days over the previous 30 days and the presence of activity limitations.

#### Mediators

##### Lifestyle behaviour habit

The 4-item automaticity subscale of self-report behavioural automaticity index will be used to assess the level of automaticity associated with physical activity, sedentary and sleep behaviours (thus 12 items in total) [81]. Example items are “Not sitting for prolonged periods is something I do without thinking”, “Consistent sleep and wake times are something I do without having to consciously remember.” Each item is assessed using a 7 point scale from Strongly Disagree to Strongly Agree. The four items for each behaviour are summed to create an overall score from 7 to 21, where higher scores indicate greater levels of automaticity. Previous research has shown that this subscale is reliable, relates to prospective behavior, and moderates between-person intention-behavior relations as theorized [81].

#### Sleep hygiene

Participants’ sleep hygiene practices will be assessed using the 13 item Sleep Hygiene Index, which assesses the frequency that participants engage in behaviours that affect sleep hygiene [82]. Each item uses a five point response scale (always, frequently, sometimes, rarely and never) which are summed to provide an overall score where higher scores indicate poorer sleep hygiene practices. The Sleep Hygiene Index has acceptable levels of internal consistency (Cronbach’s α = 0.66), test-retest reliability (*r* = 0.71), and validity compared to the Pittsburgh Sleep Quality Index (*r* = 0.37 – 0.45) and the Epworth Sleepiness Scale (*r* = 0.24).

#### Social cognitive factors

The Balanced intervention was guided by social cognitive and self-regulatory theories and items adapted from previous research are used to assess the constructs of intentions, motivation, action planning, outcome expectancies, outcome expectations, behavioural strategies, situational control, social support, and self-efficacy over a one month period [83]. The original instrument demonstrated acceptable levels of internal consistency (α = 0.63–0.79) [83]. The items were modified to align with the target behaviours of the *Balanced* intervention (e.g., regular physical activity, limit sedentary behaviour, regular sleep and wake times). A total of 11 items were used to assess these constructs for each of the three target behaviours. For each of the target behaviours a single item was used to assess intentions, motivation, action planning, situational control, social support, and self-efficacy and two items for both outcome expectancies and outcome expectations. Participants were asked to indicate their agreement with each statement using a five point scale, from Strongly Disagree to Strongly Agree.

Using five items that are answered on a five point scale, from Strongly Agree to Strongly Disagree participants reported their confidence to engage in regular physical activity, limit sedentary behaviour, and keep regular sleep and wake times, any two of these behaviours and all three of these behaviours (5 items total). Using three items that are answered on a five point scale, from Strongly Agree to Strongly Disagree participants also indicated how much they agree that their current physical activity, sedentary and sleep behaviours are a risk to their health. These items are adapted from previously used items and modified to assess the target behaviours of the intervention [49].

#### Usability, satisfaction and process evaluation

Participant perceptions of usability of the intervention platform will be assessed using the System Usability Scale, a 10 item scale that uses a 5 point response to assess agreement with each item from Strongly Agree to Strongly Disagree [84]. All items are weighted by 2.5 (including several reverse scored items) to provide an overall score from 0 to 100 where higher scores indicate greater levels of usability. Eleven items will be used to assess participant satisfaction with the intervention including the perceived usefulness of the app to self-monitor behaviours and change behaviours, the level of detailed feedback provided and the accuracy of information provided. Participants were asked to indicate their agreement with each statement using a five point scale, from Strongly Disagree to Strongly Agree. These items are similar to those used in previous studies to assess satisfaction with the intervention [28]. A randomly selected subsample of participants will also be asked to complete semi-structured interviews after completion of the study to provide information to be used as part of the process evaluation. This will include discussion on the aspects of the intervention that they believed worked well and that could be improved, information of how regularly they would like to use the app and why they did or did not use the app.

#### Engagement and use of the intervention platform

Engagement with and use of the intervention will be measured using usage statistics captured by the app database. For each behaviour and behavioural goal information is collected on the time of day and date that the entry is made and edited, the actual entry (e.g. 30 min of physical activity), and the method of entry (device-entered, user-entered). These measures are collected daily throughout the intervention period.

### Socio-demographics, Anthropometrics and cardiometabolic risk

Participants will provide information on their age, gender, years of education, occupational level, hours of work (daytime, night time, afternoon), number of days worked in previous week and average hours of work each day. Presenteeism at work over the previous 28 days will be assessed using a single item from the World Health Organization Health and Work Performance Questionnaire [85, 86]. This item asks participants to rate their performance at work on a scale of 0 to 10, where zero is the worst performance.

A research assistant blinded to group allocation will measure height, weight, waist circumference, and blood pressure with participants dressed in light clothing and without shoes. Weight (kg) will be measured on a calibrated digital scale to 0.01 kg (Biospace BSM370 Portable Automatic BMI Stadiometer, Biospace CO, Ltd., Seoul Korea). Weight will be measured twice if the two values are within 0.1 kg. If measurements vary by more than 0.1 kg a third measurement will be taken and the average of the two measures that are within 0.1 kg will be recorded. Height (cm) will be measured to 0.1 cm using a stadiometer (Biospace BSM370 Portable Automatic BMI Stadiometer, Biospace CO, Ltd., Seoul, Korea). Two measures of height will be taken and if values are not within 0.3 cm a third measure will be taken. The average of the two height measures within 0.3 cm will be taken. Waist circumference (cm) will be measured at the umbilicus (Seca 203, Seca Gmph & Co. Hamburg, Germany). Two measures will be taken, if these measures are not within 0.5 cm a third measure of waist circumference will be taken. The average of the two waist circumference measures within 0.5 cm will be taken [87].

Blood pressure and resting heart rate will be taken using a digital sphygmomanometer (Omron HEM-7320, Omron Healthcare, Co., Ltd., Kyoto, Japan) after participants have been seated quietly for 5 min. Following a 5-min minimum of sitting, two measures of blood pressure will be taken with a minimum 2-min period of rest between measures. If the two measures vary by more than 10 mm Hg (systolic), 5 mm Hg (diastolic) and 5 bpm for resting heart rate, up to five additional measures will be performed until three of the measures are within these ranges. The average of the three measures within this range will be taken [87]. Measures of total cholesterol, HDL cholesterol, LDL cholesterol, triglycerides and glucose will be taken using a capillary sample of blood drawn from the finger using the Cardiochek PA (Polymer Technology Systems, Inc., Indiana, US; BHR Pharmaceuticals Ltd., Nuneaton, UK). The measures are taken non-fasted and the time of the last meal consumed will be recorded. Capillary blood samples, presenteesism, and health related quality of life are assessed at visits 2 and 8 to limit participant burden associated with collecting capillary blood samples and due to the recall periods used in the presenteeism and health related quality of life measures.

Diet is also assessed in order to account for the effects of any changes in diet on physical activity and sleep and also measures of cardiometabolic risk [88–90]. Dietary items will assess consumption of fruit, vegetables, takeaway food consumption, drinks containing caffeine, and drinks containing alcohol. Fruit and vegetable consumption is assessed by the number of serves of these foods usually eaten each day. Consumption of takeaway food (pies, pastries, fried foods, hot chips, or takeaway meals) is assessed as the number of times per week. These items are based on an existing instrument that has demonstrated acceptable levels of validity compared to a 4 day food diary (*r* = 0.32 – 0.55) [91]. Alcohol and caffeine consumption are assessed using 4 items to assess the number of days in the last week that drinks containing alcohol and caffeine were consumed, and the number of drinks consumed on each day. These latter items are adapted from existing instrument used to assess risky alcohol consumption [92].

### Power and sample size

A statistician independent of the research team performed the power and sample size calculations were based on detecting between group differences in changes in physical activity (30 min difference, standard deviation = 30), sedentary behaviour (90 min difference, standard deviation =110), variation in time to sleep (30 min difference, standard deviation = 40), variation in time to wake (30 min difference, standard deviation = 35), and sleep efficiency (5 % difference, standard deviation = 5) at the end of the intervention. Assuming a correlation of 0.6 between the repeated measurements, a total sample of 48 participants will give the study 80 % power to detect a group by time interaction for each of the five primary outcome variables, using an alpha level of 0.01. Hence a minimum of 64 participants (32 per treatment arm) will be recruited into the study to allow for 35 % attrition over the study period.

### Analysis

Baseline data will be summarized as the number of observations, means, standard deviations, medians, minimums and maximums where the data are continuous and as number of observations and frequencies where the data are categorical. Analyses will follow an intention to treat approach. There are five primary outcomes in the study: minutes of moderate-to-vigorous intensity physical activity, minutes of sedentary behaviour, variation in time to sleep, variation in time to wake, and sleep efficiency. The primary analysis will test for between-group differences across the four study assessment points using separate linear mixed models for each outcome, with fixed effects for treatment group (device-entered vs. user-entered), time (assessment 1, 2, 3 and 4) and their interaction. Since the standard errors of the fixed effects depend on the variance-covariance structure that is used in the analysis, several possible structures including models with multiple random effects (e.g., intercept, time) as well as inclusion of autocorrelated error structures (e.g., AR1, Toeplitz) will be examined. Based on Akaike Information Criterion (AIC) the most reasonable fitting model will be selected. The interaction term will be tested at 1 % to allow for increased type 1 error rates associated with multiple primary outcomes. Secondary analyses will examine changes in composite measures of the primary outcome variables and selected secondary outcomes at the 0.05 level of significance.

## Discussion

Large proportions of the population report insufficient physical activity, high volumes of sedentary behaviour and poor sleep [8–13]. Consequently, interventions targeting these behaviours must have large reach, an objective that can be achieved by delivering interventions using technology-based approaches [26, 37]. Technology-based approaches also allow participants to access intervention materials at times and places convenient to them and do not require face to face contact which is a limitation of traditional practitioner delivered treatments e.g., behavioural change counselling [26, 37]. This study demonstrates the efficacy of a novel app-based multi-behaviour intervention to improve physical activity, sedentary behaviour and sleep quality.

An important aspect of this study is examining the relative efficacy two different approaches to self-monitoring behaviour: device-entered and user-entered. These approaches have been used previously in interventions seeking to improve physical activity and sleep, although their relatively efficacy to improve these behaviours has not been directly compared [28, 31, 93]. Examining differences in the efficacy of these self-monitoring approaches is important to inform future behaviour change interventions. Automated approaches reduce bias and burden associated with manual recording, but require resources with financial implications. It is therefore important to understand the relative efficacy on behaviour change.

Strengths of the study include a multi-behaviour approach targeting physical activity, sedentariness and sleep: this approach can leverage the potential for reciprocal effects among the three behaviours and the occurrence of any co-action effects that may occur [17, 18, 22, 90]. A further strength of the study is the recruitment of a population-based non clinical sample. Few studies have been conducted in populations who report poor sleep yet do not have a diagnosed sleep condition [17, 37], although over 20 % of the population report inadequate sleep, only approximately half of this is due to sleep conditions [94]. In addition, interventions using app-based interventions and/or website-based interventions frequently observe non-usage attrition over the first 4 weeks of the intervention period, yet do not assess behaviour change until well after non-usage attrition has occurred (e.g. 12 weeks) [28, 36, 38, 39]. A strength of this study is the timing of assessments which are intended to capture changes in behaviour and platform usage over a time period which is infrequently examined.

In summary this study simultaneously targets improvements in physical activity, sedentariness and sleep quality, and will provide important information on the efficacy of different self-monitoring strategies. A multiple behaviour intervention that can improve these behaviours and which has large reach, as provided by app-based delivery methods, has considerable potential given the health risks associated with these behaviours and the proportions of the population that engage in these behaviours in ways that adversely impact health.
